# A Case of Bilateral Carotid Body Tumors

**DOI:** 10.7759/cureus.63402

**Published:** 2024-06-28

**Authors:** Angad Pordal, Donald Morin, Rema Malik, Pritham Reddy

**Affiliations:** 1 Department of Surgery, Ascension Providence Hospital, Southfield, USA; 2 Department of General Surgery, Ascension Providence Hospital, Southfield, USA; 3 Department of Vascular Surgery, Wayne State University Detroit Medical Center, Detroit, USA; 4 Department of Vascular Surgery, Ascension Providence Hospital, Southfield, USA

**Keywords:** head and neck, radiology, carotid body, neuroendocrine tumor, carotid, vascular surgery

## Abstract

Sporadic bilateral carotid body tumors are a rare paraganglioma of the head and neck that are often asymptomatic and incidentally found during workup for other pathologies. These tumors arise from the chemoreceptor organ located at the carotid bifurcation and can be locally invasive, resulting in the involvement of adjacent neurovascular structures. With the majority of bilateral carotid body tumors having an association with familial syndromes, such as MEN2 or Von Hippel Lindau, the incidence of sporadic bilateral disease is exceedingly rare. In this case report, we report a case of bilateral carotid body tumors in a 54-year-old female with anxiety and tachycardia who underwent MRI and CTA evaluation showing evidence of left greater than right carotid body tumors. The patient was managed operatively in a staged fashion with a resolution of her presenting symptoms and no post-operative morbidity or mortality. Pathologic examination of the bilateral masses confirmed evidence of paraganglioma with immunohistochemical stains showing neoplastic cells positive for synaptophysin, chromogranin, and S100 without lymph node involvement.

## Introduction

The carotid bodies are chemoreceptors located bilaterally in the adventitia of the carotid arteries [1]. Located at the bifurcation of the internal and external carotid arteries, these small, neural crest-derived, ovoid structures play an important role in homeostasis. Carotid bodies develop from both mesodermal elements of the third branchial arch and components from the ectodermal neural crest cells [2]. These neural crest-derived components ultimately lead to the development of the glomus cells that are further differentiated into type I, which are responsible for neurotransmitter release in response to hypoxia, and type 2 cells, which are also known as sustentacular cells that support the secretory type 1 chemoreceptor [3]. In response to changes in the concentration of oxygen, carbon dioxide, and the pH of blood, these structures will release neurotransmitters to facilitate breathing and vasoresponsiveness to hypoxic or hypercarbic stimuli. Part of the paraganglioma of the body, the glomus cells of these receptors excrete neurotransmitters, such as dopamine and acetylcholine, in order to increase respiratory drive and aid in the expulsion of carbon dioxide [2]. 

While rare and only accounting for one case in 30,000 patients, these chemoreceptors may undergo excessive growth, resulting in the formation of a carotid body tumor. These tumors account for roughly 60% of paragangliomas of the head and neck [2]. With an average age of onset in the fourth to fifth decade of life and a predilection for the female gender, these slow-growing tumors are often incidentally found during workup for other complaints but may rarely produce catecholamines [1]. Furthermore, these tumors may even less frequently be bilateral, with an overall incidence of one case in 600,000 patients [2]. We report a case of a 54-year-old female without a genetic predisposition who was found to have bilateral carotid body tumors that were incidentally found and managed operatively.

## Case presentation

**Figure 1:**
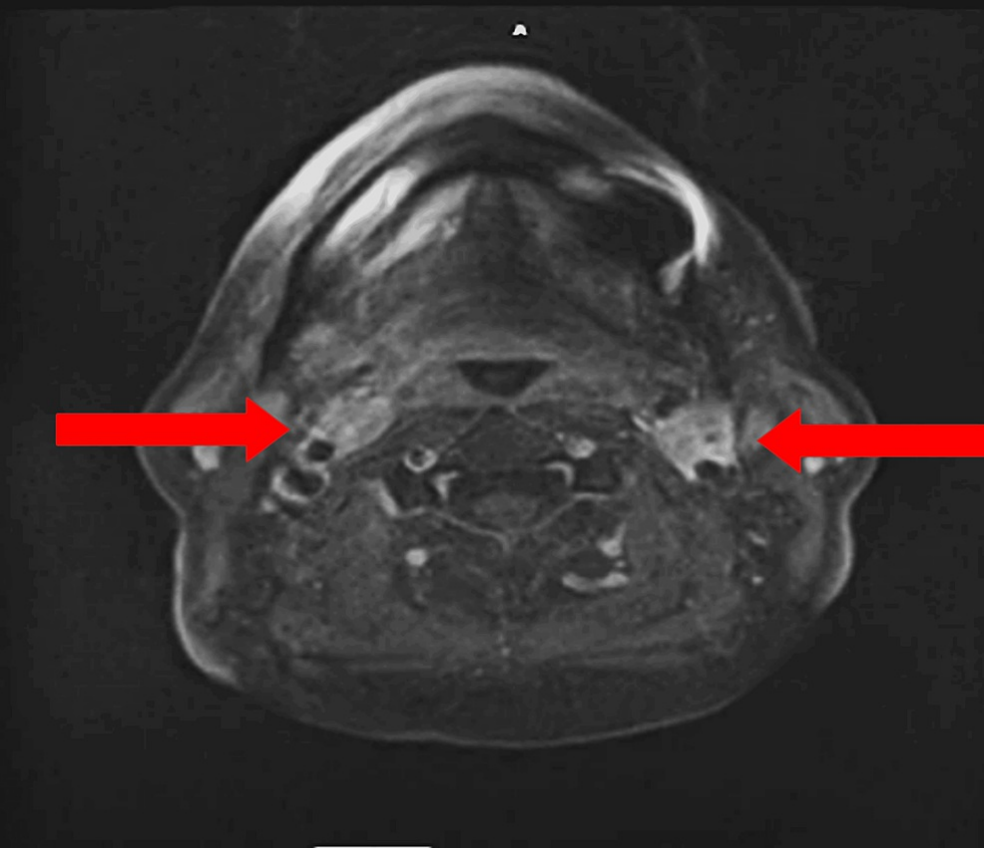
Axial T1 contrast-enhanced MRI cross-section showing enhancing bilateral carotid body tumors with splaying of the internal and external carotid arteries (red arrows)

**Figure 2:**
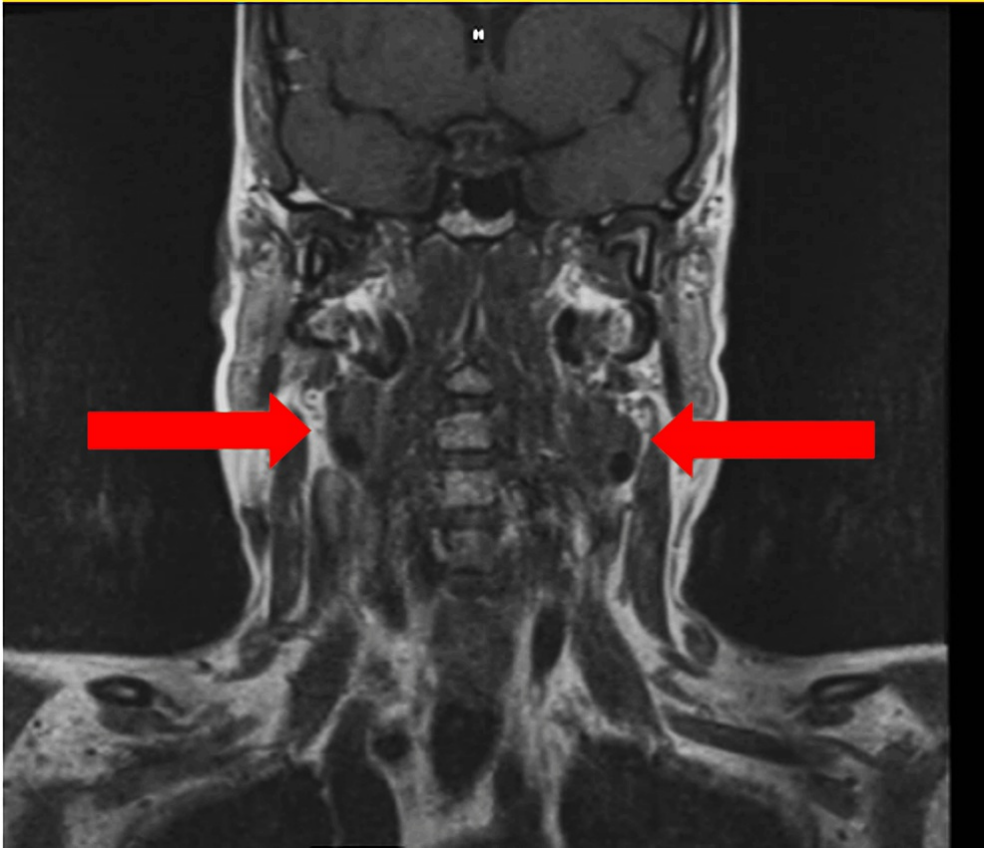
Coronal T1-weighted MRI showing bilateral carotid body tumors (red arrows) at the level of the carotid bifurcation

The 54-year-old female patient, with no past medical history, was originally seen through the emergency department for anxiety and tachycardia attributed, at that time, to panic attacks. Due to the persistence of her symptoms, the patient was subsequently seen by the neurology service as an outpatient, where MRI for further evaluation was offered to the patient. MRI of the head and neck was obtained, showing incidental evidence of a 2.4 × 1.4 × 1.4 cm left carotid mass and a 1.9 × 1.3 × 1.0 cm right carotid mass at the carotid bifurcation bilaterally (Figures 1, 2). Both masses showed T1-weighted heterogeneity and T2 hyperintensity, and the flow void was consistent with the salt-and-pepper sign. On post-contrast sequences, there will be an enhancement of carotid body tumors. Differentially, based on MRI imaging, masses were suspicious for paraganglioma versus lymphadenopathy.

**Figure 3:**
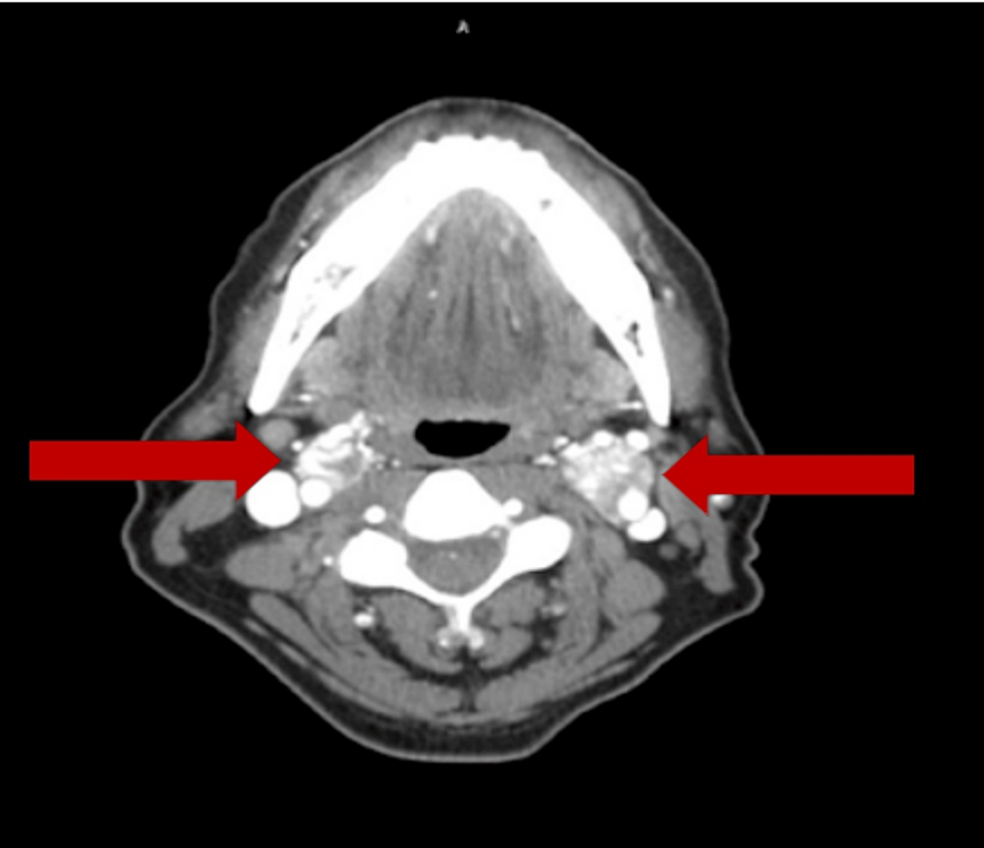
Axial CT angiogram showing bilateral heterogeneously enhancing carotid bifurcation masses (red arrows)

**Figure 4:**
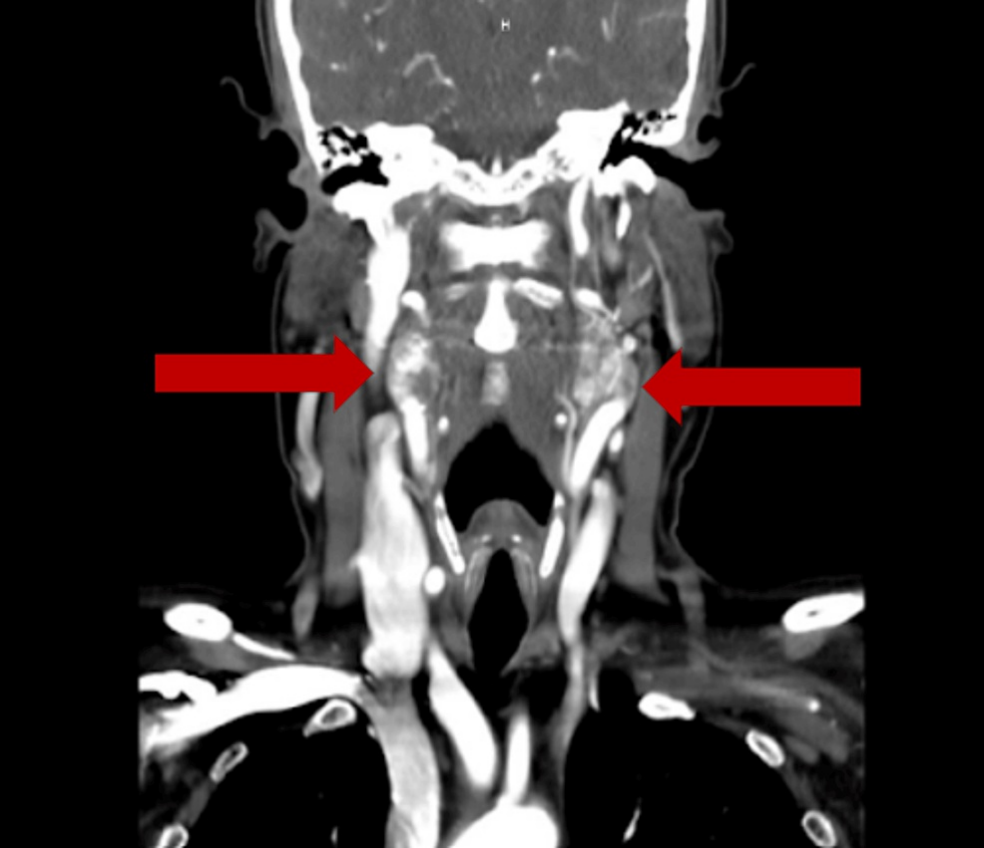
Coronal CT angiogram showing heterogenous enhancement of carotid body tumors bilaterally without significant vascular compromise (red arrows)

With a differential diagnosis concerning carotid body tumors versus lymphadenopathy but with differential considerations, including schwannoma, neurofibroma, or carotid artery aneurysm, MRI was followed by CTA in order to further delineate the etiology of these masses. CTA obtained confirmed evidence of bilateral carotid bifurcation masses, with splaying of the internal and external carotid arteries bilaterally (lyre sign). Masses were found to be well defined with intense contrast enhancement, with greater than 180 involvement of the internal and external carotid arteries bilaterally consistent with Shamblin type II. Given the focal bifurcation location and heterogeneous appearance on MRI T1, as well as the splaying of the arteries best seen on axial CTA, imaging was consistent with carotid body paragangliomas, larger on the left than the right (Figures 3, 4).

**Figure 5:**
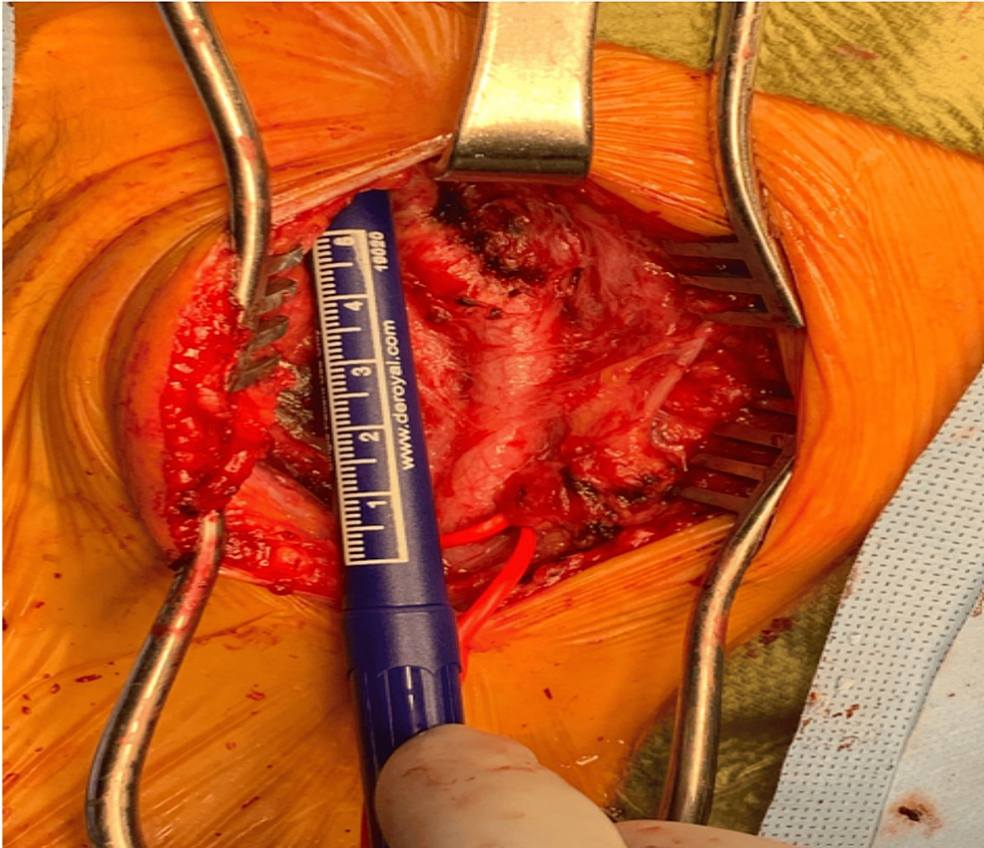
Intraoperative dissection demonstrating left internal carotid artery with removal of carotid body tumor and ligation of the external carotid artery

Given evidence of bilateral paraganglioma and increased likelihood of familial syndromes, including MEN2 and Von Hippel Lindau, cross-sectional imaging of the abdomen and pelvis was performed revealing cortical thickening of the left adrenal gland, potentially showing cortical adenoma without evidence of pheochromocytoma. Urine metanephrine studies showed no elevation of suggested pheochromocytoma. This was followed by a confirmatory serum plasma-free metanephrine and homovanillic acid testing, which also was negative. The patient was referred for genetic counseling, where a hereditary paraganglioma panel was performed using next-generation sequencing testing for several gene mutations, including SDHD, SDHA, RET, VHL, and NF1. The results of this panel were negative for germ-line mutations. The patient was seen in the vascular surgery clinic, where she was given the option of watchful waiting with serial imaging versus excision of her bilateral carotid body tumors. The patient was elected for operative intervention given her headache symptoms, which could potentially be secondary to dysregulation of her carotid bodies as well as her overall hesitancy with watchful waiting. The patient was subsequently scheduled for staged excision of her bilateral carotid body tumors to minimize potential morbidity due to concern for the degree of effacement of the tumor on the adjacent vasculature and the potential need for external carotid artery ligation for removal of the mass. The left side was chosen first, given its larger size. An oblique incision was made along the sternocleidomastoid muscle, platysma divided, and carotid sheath incised. Proximal control of the common carotid artery was obtained. The carotid body tumor was densely adherent to the carotid bifurcation. Multiple feeding branches were cauterized with the bipolar cautery. We approached the dissection from multiple angles, cranially, caudally, medially, and laterally, in order to facilitate complete mobilization of the tumor of the carotid vessels. During the procedure, care was noted to preserve the vagus, ansa cervicalis, and hypoglossal nerves. Intraoperatively, due to the adherent nature of the mass to the external carotid artery, the external carotid artery was ligated in order to provide complete resection (Figure 5).

The patient was discharged on post-operative day 1 without issue. The patient was seen and evaluated in the office two weeks post-procedure and was found to be doing well without evidence of wound issues or neurological deficits. In addition, the patient endorsed the resolution of her headache and tachycardia symptoms. Subsequently, three weeks after the index operation, the patient was brought back for excision of the contralateral (right) side in a similar fashion. Due to the smaller size of the right carotid body tumor and less adherent nature, the external carotid artery was spared. The patient progressed well post-operatively and was once again discharged on post-operative day 1. The patient was seen in the clinic for two-week and eight-week follow-ups and was found to be without complaints of motor or sensory neurological deficits. No additional cross-sectional imaging was obtained post-operatively due to the lack of neurological or local site complications. Pathology of the bilateral masses excised confirmed evidence of paraganglioma with immunohistochemical stains showing neoplastic cells positive for synaptophysin, chromogranin (weak), and S100 (around sustentacular cells), supporting the diagnosis of paraganglioma.

## Discussion

Carotid body tumors are rare paragangliomas emanating from parasympathetic neural crest cells, accounting for 50-60% of all head and neck paragangliomas but only 0.5% of all head and neck tumors [4]. These non-functional tumors are rarely implicated (less than 1%) with the catecholamine secretion associated with sympathetic paragangliomas, such as adrenal pheochromocytomas [5]. These tumors have a broad distribution, with the average age of onset being within the fifth and sixth decade of life [6]. While presentation in adolescence is rare, familiar variants are often found in the fourth decade of life [6]. All forms, whether sporadic, hyperplastic, or familiar, have been found to have increased prevalence in females compared to males, with a 2:1 increased likelihood [6]. The carotid body is a chemoreceptor responsible for identification of cerebral hypoxia, hypercapnia, and acidosis. It has been shown that chronic hypoxia is a risk factor for the development of carotid body tumors, as seen in the 8:1 increase in incidence in those who are residents of high-altitude locations [7].

Several gene pathways have been identified in the development of carotid paragangliomas, including RET, NF1, VHL, and succinate dehydrogenase (SDH) [4]. SDH plays a key role in the enzymatic function of the mitochondrial electron transport chain with alterations in the SDHA, SDHB, SDHC, and SDHD proteins of SDH, resulting in increased development of carotid paragangliomas [8]. There are three major categories of carotid body tumors, such as hyperplastic, familial, and sporadic, with the majority (85%) being sporadic. The majority of the sporadic forms are unilateral, with only 5% reported in the literature to be bilateral [9]. Familial forms are ~10%, with 30% of those noted to be bilateral [4]. The most commonly associated syndrome with carotid body tumors is paraganglioma syndrome 1 (PGL1). This syndrome is caused by SDHD mutations on Chr 11q23.1. Approximately 80-90% of individuals with PGL1 will have multiple paragangliomas. In addition, functional paragangliomas such as pheochromocytoma occur in up to 40-50% of patients with PGL1 [4]. Carotid bodies are often highly vascularized, with their blood supply from the ascending cervical branch of the external carotid artery. These lesions splay the carotid bifurcation as they grow, but the narrowing of the internal or external carotid arteries as a result of tumor growth is rare. These tumors are benign and slow-growing and often asymptomatic. Their slow-growing nature makes it such that presentation may be delayed with bruit and thrill as rare late physical exam findings, with a neck mass and symptoms such as hoarseness, dysphagia, and cranial nerve palsies [5]. CT angiography is the gold standard and will reveal a hyper-vascular mass with significant tumor blush, feeding arteries, and draining veins [7]. Splaying of the internal and external carotid arteries, known as the lyre sign, can be seen on cross-sectional imaging, confirming the location of the mass at the carotid bifurcation and supporting the diagnosis of carotid body tumor [7]. MRI imaging may be obtained during the workup of the cervical mass and will show a salt-and-pepper appearance, representing the bright hyperintense hemorrhage (salt) with corresponding black flow voids (pepper) [10]. As with other neuroendocrine tumors, octreotide nuclear scan has also been shown to be effective in the identification of carotid body paraganglioma. Its utility is greatest in the determination of residual disease versus scar post-operatively [8].

The Shamblin classification is widely adopted as a classification system to describe the anatomy of these tumors. Type 1 tumors are localized within the carotid vessels, type 2 tumors partially adhere or surround the carotid arteries, and type 3 tumors fully surround at least one of the carotid vessels. Due to the hypervascular nature of these tumors and the proximity of nearby neurovascular structures, biopsy for diagnosis is contraindicated because of the unacceptable risk of hemorrhage, pseudoaneurysm, or carotid thrombosis [11]. The treatment involves surgical resection, which has been previously shown to have a low neurovascular morbidity and overall mortality rate. Once diagnosed, excision is recommended to prevent an increase in size, which may confer greater operative risk. Operative approaches are most often performed with the anterior sternocleidomastoid incision. In patients with bilateral disease, the approach is staged in order to prevent intraoperative hemodynamic instability from the excision of the bilateral nerve of Hering excisions [12]. For those patients who are unable to tolerate surgery or if operative intervention is not an option, radiotherapy is an alternative treatment modality that can cease the tumor growth or decrease its size [13].

## Conclusions

Bilateral carotid body tumors are a rare paraganglioma of the head and neck arising from the chemoreceptors at the bifurcation of the carotid arteries. With three major sub-categories and most bilateral tumors being associated with familial syndromes, workup including evaluation for MEN2 and Von Hippel Lindau is necessary. Diagnosis is often made based on CT angiogram findings, with MRI being a useful adjunct. Management of carotid body tumors involves operative excision of the mass with or without ligation of the external carotid artery, taking care to avoid adjacent neurovascular injury. In this case report, we present a rare case of sporadic bilateral carotid body tumors found incidentally in a 54-year-old female who underwent staged bilateral excision without operative morbidity or mortality.
